# Supplementary material for: The interactome of intact mitochondria by cross-linking mass spectrometry provides evidence for coexisting respiratory supercomplexes
Source: Mol Cell Proteomics. 2017 Dec 8;17(2):216–32. doi: 10.1074/mcp.RA117.000470 (PMC5795388; doi:10.1074/mcp.RA117.000470)
Supplement: Supplemental Data [file supp_RA117.000470_133922_0_supp_23978_fzffwf.zip › spectra_annotation/mito_spectra_annotation/497-1-8-1-4-1.pdf]

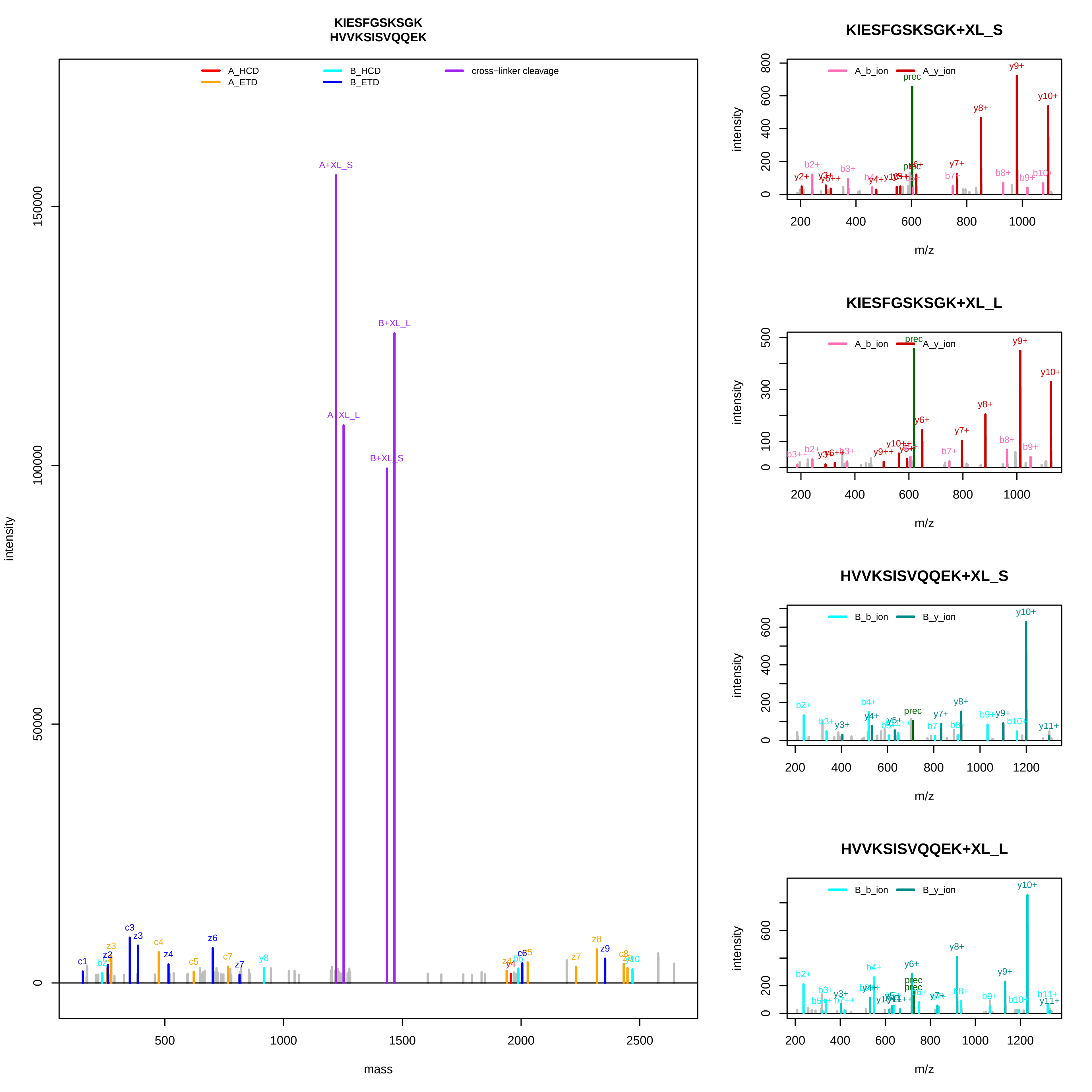
KIESFGSKSGK
HVVKSISVQQEK
A_HCD
A_ETD
B_HCD
B_ETD
cross−linker cleavage
A+XL_S
B+XL_L
A+XL_L
B+XL_S
c1
z2
z3
c3
z3
c4
z4
c5
z6
c7
z7
y8
z7
z8
z9
c8
y10
intensity
mass
0
50000
100000
150000
500
1000
1500
2000
2500
KIESFGSKSGK+XL_S
A_b_ion
A_y_ion
prec
y9+
y10+
y8+
y7+
intensity
m/z
KIESFGSKSGK+XL_L
A_b_ion
A_y_ion
y9+
y10+
y8+
y6+
y7+
intensity
m/z
HVVKSISVQQEK+XL_S
B_b_ion
B_y_ion
y10+
y8+
b4+
b2+
prec
y11+
intensity
m/z
HVVKSISVQQEK+XL_L
B_b_ion
B_y_ion
y10+
y8+
y6+
b4+
b2+
y9+
intensity
m/z
